# Zam Is a Redox-Regulated Member of the RNB-Family Required for Optimal Photosynthesis in Cyanobacteria

**DOI:** 10.3390/microorganisms10051055

**Published:** 2022-05-20

**Authors:** Patrick E. Thomas, Colin Gates, William Campodonico-Burnett, Jeffrey C. Cameron

**Affiliations:** 1 Department of Biochemistry, University of Colorado, Boulder, CO 80309, USA; patrick.thomas@colorado.edu (P.E.T.); will.campo@colorado.edu (W.C.-B.); 2 Renewable and Sustainable Energy Institute, University of Colorado, Boulder, CO 80309, USA; cgates4@luc.edu; 3 National Renewable Energy Laboratory, Golden, CO 80401, USA

**Keywords:** reduction-oxidation, photosynthesis, cyanobacteria, photosystem II, phycobilisome, RNase, light-harvesting

## Abstract

The *zam* gene mediating resistance to acetazolamide in cyanobacteria was discovered thirty years ago during a drug tolerance screen. We use phylogenetics to show that Zam proteins are distributed across cyanobacteria and that they form their own unique clade of the ribonuclease II/R (RNB) family. Despite being RNB family members, multiple sequence alignments reveal that Zam proteins lack conservation and exhibit extreme degeneracy in the canonical active site—raising questions about their cellular function(s). Several known phenotypes arise from the deletion of *zam*, including drug resistance, slower growth, and altered pigmentation. Using room-temperature and low-temperature fluorescence and absorption spectroscopy, we show that deletion of *zam* results in decreased phycocyanin synthesis rates, altered PSI:PSII ratios, and an increase in coupling between the phycobilisome and PSII. Conserved cysteines within Zam are identified and assayed for function using in vitro and in vivo methods. We show that these cysteines are essential for Zam function, with mutation of either residue to serine causing phenotypes identical to the deletion of Zam. Redox regulation of Zam activity based on the reversible oxidation-reduction of a disulfide bond involving these cysteine residues could provide a mechanism to integrate the ‘central dogma’ with photosynthesis in cyanobacteria.

## 1. Introduction

Cyanobacteria are photosynthetic prokaryotes that play major roles in the biosphere—serving as the major fixers of nitrogen in the ocean and as primary producers [1]. As their ancestors gave rise to modern chloroplasts, cyanobacteria serve as model systems for both carbon fixation and oxygenic photosynthesis. Cyanobacteria are also considered a promising chassis for metabolic engineering due to their diverse metabolisms [2]. While decades of research have elucidated processes by which cyanobacteria regulate gene expression and metabolism, the mechanisms underlying many regulatory processes remain unknown. However, there is growing evidence that in photosynthetic organisms, redox regulation plays a crucial role in linking the cellular energy state with downstream regulatory networks involved in gene expression and RNA turnover.

The resistance to acetazolamide (*zam*) gene was first identified in the cyanobacteria *Synechocystis* sp. PCC 6803 (hereafter PCC 6803) [3]. Either a deletion or an insertion mutation in *zam* confers resistance to lethal concentrations of the carbonic anhydrase inhibitor acetazolamide to PCC 6803 [4]. Zam was also identified as a redox-responsive protein in both PCC 6803 and *Synechococcus* sp. PCC 7002 (hereafter PCC 7002) in two separate redox-proteomics experiments [5,6]. It was initially presumed that *zam* encoded a transporter which affected the accessibility of acetazolamide to one or more of the cellular carbonic anhydrases; however, multiple sequence alignments show that Zam is actually a member of the RNase II/R family [4,7]. Redox-based control of RNA modulating enzymes is not unprecedented in cyanobacteria. For example, the expression of the cyanobacterial RNA helicase gene, *crhR*, is controlled by the cellular redox state and photosynthetic electron transport chain [8]. The CrhR protein, important for cold-tolerance, interacts with multiple RNA species involved in photosynthesis and the degradosome, suggesting a regulatory feedback loop [9,10,11].

RNase II/R proteins are, with few exceptions, 3′-5′ exonucleases with multiple roles in RNA degradation and processing [12,13]. These proteins often work in collaboration with other nucleases and have been found to be part of the degradosome, a multi-protein complex for degrading RNA, in diverse bacterial systems [14,15,16]. The catalytic activity of these proteins comes from the RNase II domain (RNB), the active site of which is essentially invariant across all three domains of life [12,17,18,19]. The best studied RNB proteins in prokaryotes are the eponymous *E. coli* RNase II (*E. coli* Rnb) and *E. coli* RNase R (*E. coli* Rnr). At the protein level *E. coli* Rnb and *E. coli* Rnr share 60% amino acid sequence identity and have identical domain architecture, with the only unique sequence level feature of the proteins being a long basic region at the C-termini of *E. coli* Rnr that is not found in *E. coli* Rnb [19]. Despite these similar structures, *E. coli* Rnr and Rnb exhibit unique catalytic activities; Rnr is capable of digesting through structured regions of RNA while Rnb is not [19,20,21]. While Zam proteins are clearly RNase II/R family proteins, whether they are RNase II or RNase R type enzymes is currently unknown.

Due to the similarities between RNase II type and RNase R type enzymes, identification of homologs based on amino acid sequence identity alone is difficult and additional biochemical tests with purified enzymes are often necessary [19,22]. Cyanobacteria encode two RNB proteins on their chromosome, one of which is typically annotated as Zam; some species also encode RNB homologs on endogenous plasmids [7]. Here we use RNB domain protein sequences from multiple cyanobacterial genomes to gain insight into the evolutionary history of Zam and its place in the larger RNase II/R family. Observations based on our sequence analysis led us to multiple hypotheses about the function of Zam, which we tested using various physiological and biophysical assays.

## 2. Materials and Methods

### 2.1. Reciprocal Best Hit, Multiple Alignment, and Phylogenetics

Protein sequences of the two RNB-containing proteins from PCC 7002_A0574 (Rnb) and PCC_A1543 (Zam) were downloaded from the National Center for Biotechnology Information (https://www.ncbi.nlm.nih.gov/, accessed on 28 February 2019). The sequences were used as query sequences to search for homologs within a select set of diverse cyanobacterial genomes using NCBI BLAST [23]. Top hits from each genome were then used as BLAST query sequences against the PCC 7002 genome to identify reciprocal best hits (RBH) for Zam and Rnb for each species. Each RBH was considered a putative ortholog of either Zam and Rnb and are designated as such throughout this work.

Sequences of Zam or Rnb homologs were downloaded using batch ENTREZ on 28 February 2019. Multiple sequence alignment was performed using the Clustal-Omega algorithm through the EMBL server. Sequences of Zam and Rnb homologues were pooled and aligned with the sequences of *E. coli* Rnr, *E. coli* Rnb, and both chloroplastic and nuclear RNB sequences for *C. reinhardtii.* MEGA-X was used to generate a bootstrapped, maximum likelihood, and consensus tree based on 500 replicates.

### 2.2. Domain Identification

The sequence of each Zam ortholog was input into the EMBL-HMMER protein domain search tool using the default parameters and results were downloaded. Domain maps were created to scale using Adobe Illustrator.

### 2.3. Homology Mapping and Visualization

Multiple alignments were performed as described above for the Zam ortholog group or the Rnb ortholog group with the sequence of *E. coli* Rnr present in each group. For both alignments, in UCSF-Chimera an alignment was associated with the crystal structure of *E. coli* Rnr (PDB:5XGU) and the percent conservation at every residue was mapped onto the structure [21,24].

### 2.4. Computation Modeling

Threading was performed using the iterative threading assembly refinement (I-TASSER) server [25]. The sequence of Zam was uploaded using the default parameters which allowed the software to identify the best template crystal structure available in the protein database (PDB). *E. coli* Rnr was identified by the algorithm as the best match, and a structural model based on this was output accordingly.

### 2.5. Cell Growth and Maintenance

PCC 7002 strains were grown under constant illumination (~150 μmol photons m^−2^ s^−1^) by cool white fluorescent lamps at 37 °C in AL-41 L4 Environmental Chambers (Percival Scientific, Perry, IA, USA). Cultures were grown either in liquid A+ medium in 125 mL baffled flasks closed with foam stoppers (Jaece Identi-Plug), or on A+ media solidified with Bacto Agar (1%; *w*/*v*) [26]. Antibiotics (kanamycin, 100 μg/mL; spectinomycin, 100 μg/mL) were used to grow strains with appropriate resistance cassettes.

### 2.6. Plasmid and Strain Construction

The plasmid used for *zam* deletion (pJCC254) was supplied by Jeffrey Cameron for use in this work [7]. All other plasmids for gene insertion and protein expression were cloned using Gibson Assembly [27]. Assembled plasmids were made using various backbones and inserts amplified by PCR using Phusion polymerase (Thermo Fisher Scientific, Waltham, MA, USA). After assembly, plasmids were transformed, isolated, and sequenced using standard molecular biology protocols.

Cyanobacterial strains were created by transformation through homologous recombination. Exponentially growing cultures were inoculated with 0.5–2 ng/mL of plasmid containing a desired insert flanked by 500 base pair homology arms. Cells and plasmid were incubated in 1.7 mL microfuge tubes overnight at constant illumination at 37 °C without shaking. After incubation, transformants were selected using appropriate antibiotic either on a pre-warmed A+ agar plate or in liquid A+ shake flasks. After initial selection, potentially positive transformants were streaked on plates, isolated after growth, and verified by colony PCR. Positive colonies were passaged twice on antibiotic containing media and then checked for segregation. Insert regions within segregated colonies were amplified by PCR and products were sent for sequencing to verify that the insert carried the correct sequence.

### 2.7. Spot Plates

PCC 7002 cells of each line were taken from freshly grown plates and used to inoculate 50 mL of liquid A+ supplemented with appropriate antibiotics. This primary preculture was grown overnight. After growth, precultures were diluted in new flasks to OD_750nm_ = 0.05 in A+ without antibiotics and allowed to grow overnight. Dilution and growth were repeated to ensure that there was no antibiotic present in the media. Final precultures were diluted to OD_750nm_ = 0.05. The diluted precultures were then used to make 10-fold serial dilutions. Spots (5 µL) of each dilution were placed on an A+ agar plates and allowed to dry for 30 min before being placed in 37 °C incubator.

### 2.8. Nitrogen Starvation and Repletion

Three flasks of each cell line used were precultured as described for spot plates. Final precultures were pelleted at 4300× *g* for 15 min. Cells were washed twice by resuspension and pelleting with A+ -NO_3_. After washing cells were resuspended in A+ -NO_3_ and diluted to OD_750nm_ = 0.2. Cells were then grown under standard conditions as described above. Triplicate samples were taken from each flask at multiple timepoints and absorption spectra recorded using a Tecan plate reader with a 5 nm step size from 400 nm to 750 nm. After 30 h, when the phycocyanin peak was gone, the cells in each flask were pelleted, resuspended in fresh A+ -NO_3_, and diluted to OD_750nm_ = 0.2 using A+ -NO_3_. After cells were appropriately diluted, 500 mL of filter-sterilized 100 g/L NO_3_ was added to each flask. Flask were placed back in the incubator and grown as during starvation. Samples were taken as during starvation.

### 2.9. 77 K Fluorescence Spectroscopy

Each cell line used was precultured as described for spot plates. The final preculture was inoculated at OD_750nm_ = 0.05 and allowed to grow overnight. Cells were concentrated 5× in 1.7 mL microfuge tubes by low speed centrifugation with a clear top microfuge under constant illumination. A 0.5 mL quantity of each cell line was removed and placed in NMR tubes. NMR tubes with cells were placed back in the incubator under light conditions identical to growth for 30 min. The remaining 1 mL of sample was pelleted by centrifugation at 18,000× *g*.

Following this, 77 K fluorescence was performed on a Fluorolog-3 spectrofluorometer (Horiba, Kyoto, Japan) with a liquid nitrogen Dewar attachment to maintain sample temperature. Samples in NMR tubes were removed from light immediately before flash freezing in liquid nitrogen. Fluorescence emission spectra were collected from frozen samples following the excitation of chlorophyll (440 nm excitation; 600–800 nm emission) or phycocyanin (580 nm excitation; 600–800 nm emission). Data from chlorophyll excitation were normalized to the PSII emission maximum (692 nm) and data from phycocyanin excitation were normalized to PSI emission maximum (712 nm).

### 2.10. Quantitative Long-Term Time-Lapse Fluorescence Microscopy

Cells in late-exponential or linear growth were mixed together based on equal OD_750 nm_ and spotted on A+ medium solidified with 1.5% Bacto agar. Cells were equilibrated at 37 °C for 30 min in the dark prior to imaging using a custom Nikon TiE microscopic system and transilluminated growth light provided at 640 nm (50% power) as previously described [28]. Fluorescence of Chl-a and phycobilisomes were monitored following specific excitation using solid-state light sources at 640 nm or 555 nm (SpectraX, Lumencore, Beaverton, OR, USA). Emission of Chl-a and phycobilisome fluorescence was collected through standard Cy5 or RFP filter cubes (Nikon, Tokyo, Japan), respectively.

### 2.11. Protein Expression and Purification

*E. coli* (BL21-DE3) was transformed with a plasmid containing the coding sequence for MBP-*zam*-6xHis under a lactose inducible promoter. Transformants were selected on LB agar plates supplemented with kanamycin (50 µg/mL). Plates were kept at 37 °C overnight. A single colony was picked using a sterile toothpick, transferred into LB supplemented with kanamycin (50 µg/mL), and grown with shaking (200 rpm) for 6 h at 37 °C. The culture was then split, diluted 1:100 into fresh LB with kanamycin, and grown overnight to serve as starter cultures for expression.

Overnight cultures were used to inoculate (1:100) 5 L baffled flasks containing 600 mL pre-warmed LB supplemented with kanamycin (50 µg/mL) and glucose (0.2% *w*/*v*). Cells were grown ~3 h until OD_600nm_ = 1.0, and then induced with 1 mM isopropyl β- d-1-thiogalactopyranoside (IPTG). Cells were harvested after 3 h by pelleting in a Beckman Coulter Allegra X-14R Centrifuge with a SX4750A rotor at 4300× *g*, 4 °C for 10 min. Cell pellets were flash frozen with liquid nitrogen and kept at −70 °C overnight. Cells were thawed on ice and resuspended in lysis buffer (20 mM Tris pH 7.9, 200 mM KCl, 1 mM MgCl_2_, 10% glycerol) supplemented with 1× Halt protease inhibitor (ThermoFisher), 1 mM PMSF (phenylmethylsulfonyl fluoride), 1 mg/mL lysozyme, 0.01% TWEEN 20 (Sigma, St. Louis, MO, USA), 6.25 U/mL Benzonase, and 10 mM 2-mercaptoethanol (βME). The resuspended pellet was incubated on ice for 10 min and lysed via sonication on ice with a Qsonica Q55 Sonicator Ultrasonic Homogenizer with Probe 55 W (10× pulses, 30 s on, 30 s off, 100% amplitude). Lysate was clarified by two rounds of centrifugation at 11,000× *g* for 15 min. Clarified lysate was passed through a 0.45-micron filter to remove any additional clumps.

Lysate was mixed with Ni-NTA bead slurry (Thermo Fisher) which had been prepared for binding by preincubation with lysis buffer and incubated with agitation for 1 h at 4 °C. After incubation, the lysate/bead slurry were separated by flowing the mixture over a gravity column. Flow through was collected. Beads were then washed/eluted with lysis buffer containing progressively higher concentration of imidazole (10–500 mM). Elution samples were assayed for presence of protein via SDS-PAGE (Figure S1). Samples containing protein were pooled to be used for MBP-purification.

Pooled eluate from the nickel column was diluted 1:10 in lysis buffer and flowed over an amylose resin (New England Biolabs, Ipswich, MA, USA) gravity column prepared according to the resin manufacturer’s specifications. Flow-through was collected. Amylose resin was washed with lysis buffer and then protein was eluted from column using lysis buffer supplemented with 10 mM maltose. Fractions were assayed by SDS-PAGE. Positive fractions were pooled and dialyzed overnight at 4 °C in 1× binding buffer.

### 2.12. Analysis of CSD1 RNA Binging Potential

RNase II residues involved in binding were extracted from the literature and the RNase II-RNA bound crystal structure (PDB: 2IX1). Our Zam protein multiple alignment was uploaded to the UC-Berkeley WebLogo site and a sequence logo was generated to enhance visualization of conserved residues [29]. Conserved aromatic and positively charged residues were manually identified from the sequence logo and matched to their corresponding *E. coli* residues for evaluation. The positioning of secondary structure elements was performed using the threaded structure of Zam generated by I-Tasser.

## 3. Results

### 3.1. Domain Architecture and Conservation of Zam in Cyanobacteria

**Figure 1:**
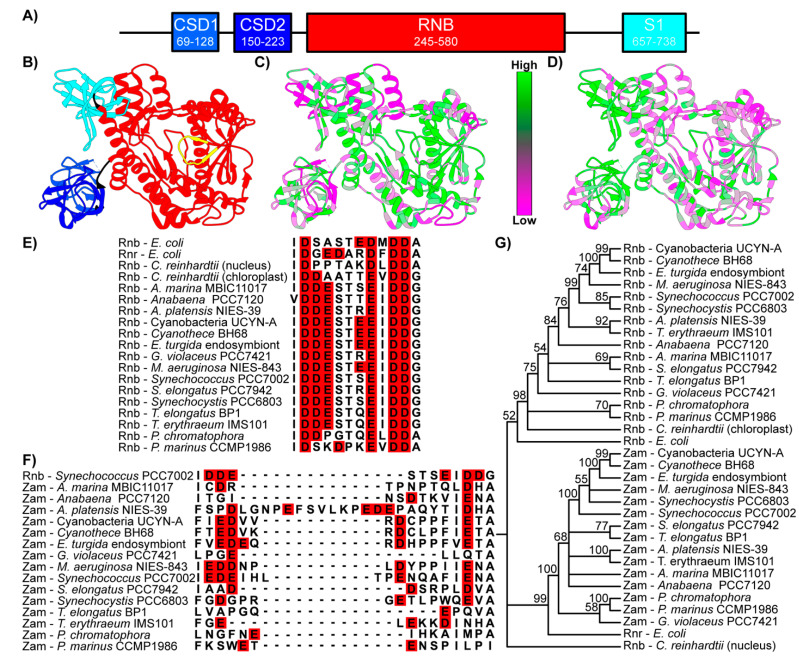
**Domain architecture and conservation of Zam.** (**A**) Map of PCC 7002 Zam domains as identified by Pfam. Each predicted domain of Zam corresponds to a domain found in *E. coli* Rnr. (**B**) *E. coli* Rnr crystal structure with domains color coded to match Zam domain map. The active site shown in alignments is indicated in yellow. (**C**) Cyanobacterial Rnb protein conservation mapped to *E. coli* Rnr crystal structure. Conservation is observed across the protein but is most concentrated around the RNB domain active site. (**D**) Cyanobacterial Zam conservation mapped onto *E. coli* Rnr crystal structure. Most conservation is observed in the RNA binding domains (CSD1, CSD2, S1). Low conservation is observed around the active site. (**E**) Multiple alignment of cyanobacterial Rnb protein active sites with outgroups shown as reference. Aspartic and glutamic acid residues color coded in red. (**F**) Multiple alignment of *zam* protein active sites. Aspartic and glutamic acid residues color coded in red. Active site regions were identified by alignment with active site region shown in Figure 1E. The sequence of Rnb from PCC 7002 is included to represent functional RNB domain active sites. (**G**) Bootstrap consensus tree of cyanobacterial Rnr, cyanobacterial Rnb, and outgroup homolog protein sequences. Cyanobacteria Rnb genes cluster as a distinct group along with chloroplastic Rnb genes and the *E. coli* Rnb gene. Zam proteins form their own cluster with *E. coli* Rnr. An Rnb domain containing protein from the nuclear genome of *C. reinhardtii* is provided as an outgroup.

Comparative sequence and structural analysis was first performed to gain insight into the function of Zam. Pfam analysis shows that in addition to the RNB domain that defines the RNase II/R protein family, Zam also contains two cold-shock domains and an S1 domain (Figure 1A) and exhibits structural similarity with *E. coli* Rnr (Figure 1B). Both S1 and cold-shock domains are members of the larger OB-fold family and have been implicated in RNA binding, carbohydrate binding, and protein–protein interactions in multiple systems [30].

Given the similarity of the domain architecture within the Zam proteins, we wondered if there was extensive primary sequence conservation in any regions that might indicate shared function. To identify regions of interest, the percentage of amino acid conservation calculated from multiple sequence alignments of the cyanobacterial Rnb homologs (Figure 1C) or Zam homologs (Figure 1D) was mapped onto the *E. coli* Rnr crystal structure [21] using Chimera [24]. The Rnb homolog conservation was to serve as a control for general patterns of conservation seen across RNase II/R family proteins. However, patterns of conservation between the two protein groups were strikingly different. In the Zam proteins, conservation is clustered primarily around the three OB-fold containing domains (Figure 1D). In contrast, conservation is more limited in the OB-fold containing domains of Rnb proteins and is mostly found in the RNB nuclease domain (Figure 1C).

Comparing primary sequences within the RNB domain explains the stark conservation difference. The active sites of the Rnb proteins (Figure 1E) and the Zam proteins (Figure 1F) reveal that, while the canonical RNB domain is present in cyanobacteria, the Zam proteins have highly degenerate and poorly conserved ‘active’ sites. Most Zam proteins lack the four aspartic or glutamic acid residues required for coordination of the Mg^2+^ that is essential for catalysis [31,32]. These acidic residues are essentially invariant in all known functional RNase II/R proteins, suggesting that all Zam proteins lack nuclease activity, but could retain RNA-binding activity.

### 3.2. Zam and Rnb Phylogeny

Using BLAST, we identified reciprocal best hits (RBH) for the annotated Zam and Rnb proteins from our model species *Synechococcus sp.* PCC 7002 in a diverse set of cyanobacterial genomes. We also searched for homologs to Zam and Rnb in the genomes of other photosynthetic organisms. Zam-like proteins were not found in any algae or higher plant genome queried. Interestingly, chloroplast genomes do contain an RBH for Rnb, but lack Zam. While many RNB domains containing proteins were identified in all species whose genomes were searched, an RBH for Zam was only found in *Paulinella chromatophora* and *Epithemia turgida*. Both *P. chromatophora* and *E. turgida* have experienced recent endosymbiotic events [33,34] and their Zam proteins are thus cyanobacterial in origin.

A phylogenetic tree for representative Zam and Rnb proteins was created (Figure 1G). As expected, the Rnb proteins cluster into their own clade, along with the chloroplastic nuclease from *C. reinhardtii*, and *E. coli* Rnb. This supports the idea that these proteins are genuine RNase II proteins. The Zam proteins also cluster into their own clade with *E. coli* Rnr. This clear divergence into distinct protein families, along with the presence of a Zam protein in every cyanobacterial genome searched, indicate that the Zam and the Rnb proteins diverged early in the cyanobacterial lineage, and that Zam is most likely a homolog of *E. coli* Rnr.

### 3.3. Zam Proteins Possess Conserved, Redox-Active Cysteines in CSD1

**Figure 2:**
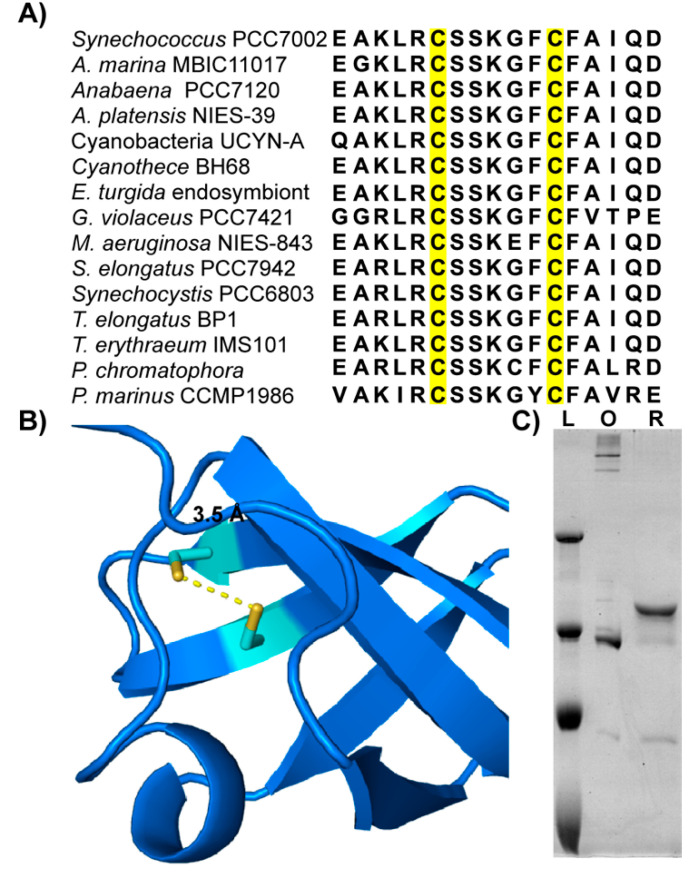
**Identification of Redox-Active Cysteines in Zam:** (**A**) Multiple sequence alignment of cyanobacterial Zam CSD1 domain focused on the CSD1 OB-fold. Conserved cysteines are highlighted in yellow. (**B**) Threaded structure prediction of PCC 7002 Zam CSD1 domain with conserved cysteines indicated in yellow. (**C**) SDS-PAGE of purified PCC 7002 Zam in oxidizing (O) and reducing (R) conditions. A quantity of 1.3 µM protein was incubated in 10 mM oxidized glutathione and then was diluted 1:20 in buffer without glutathione (oxidized) or in the presence of 710 mM βME (Reduced) and incubated at room temperature for 1 h. The samples were boiled for 5 min prior to loading. Ladder (L) shows apparent molecular weights (250 kDa, 130 kDa, 100 kDa, 70 kDa). Reduced protein runs at an apparent higher molecular weight.

Given the extreme divergence of putative Zam active site regions, we chose to investigate the regions that were conserved. The first cold shock domain (CSD1) of Zam is highly conserved among homologs (Figure 1D). Of special interest in this domain are two invariant cysteines (C73 and C79 in PCC 7002) (Figure 2A) that were previously identified as reduction-oxidation (redox) active in global redox-proteomics studies of two different cyanobacterial species [5,6].

Due to the proximity of these cysteines to one another and the well-recognized role of disulfide bond formation in the redox regulation of proteins, we built a model of CSD1 to assess whether its conserved cysteines could potentially form a disulfide bridge [35]. With the *E. coli* Rnr crystal structure as a reference, threading was performed to create a model of PCC 7002 Zam. The modeled structure places these cysteines less than 4 Angstroms apart in an orientation favoring disulfide bond formation (Figure 2B).

While computational modeling and proteomics studies support the hypothesis that the conserved cysteines in Zam could undergo disulfide bond formation, we wanted to validate that PCC 7002 Zam was redox responsive in vitro. In *E. coli*, we expressed PCC 7002 Zam fused to an N-terminal maltose binding protein and a C-terminal 6x-histidine tag (Figure S1). The recombinant Zam was purified and analyzed by SDS-PAGE under both reducing and oxidizing conditions (Figure 2C). The mobility of recombinant Zam is influenced by the redox state of the protein, with the reduced form of the protein running at an apparent higher molecular weight when compared to the oxidized form. This result is consistent with a protein capable of undergoing reduction of cysteines and leads us to conclude that Zam likely forms a disulfide bond [36]. Further evidence for disulfide bond formation comes from recent structural predictions of Zam from PCC 6803 using AlphaFold [37,38]. The predicted 3D structure (https://alphafold.ebi.ac.uk/entry/Q46363, accessed on 13 May 2022) includes a disulfide bond between C73 and C79 and a per-residue confidence score (pLDDT) of greater than 90 at these residues, indicating a very high model confidence.

### 3.4. Analysis of CSD1 RNA Binding Potential

OB-fold domains are known to bind to numerous different ligands including single-stranded DNA, RNA, and proteins [30]. In other members of the RNase II/R family, the CSD1 OB-fold is known to bind RNA in in vitro studies and to contact RNA in crystal structures [32,39,40]. In the *E. coli* sRNase II co-crystalized RNA–protein complex, the major contact point between CSD1 and RNA occurs at the loop between the first and second β-strand (L12) of CSD1 [32]. The contacts in L12 of the *E. coli* protein appear to be mediated by four residues—two lysine residues and two phenylalanine residues, which are commonly used in the recognition of ssRNA [32].

**Figure 3:**
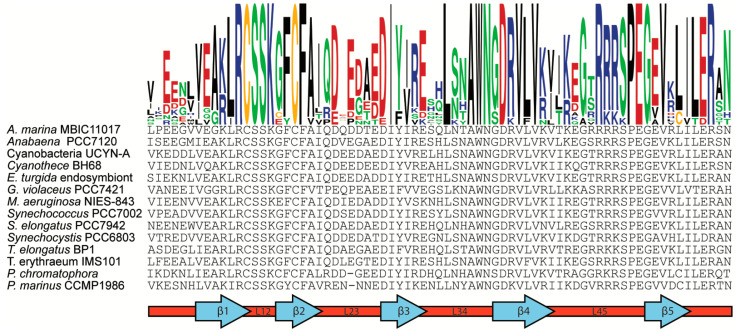
**Annotated alignment of CSD1 OB-fold domain from Zam proteins.** Conservation is illustrated with a sequence logo colorized by amino acid class (Black = Hydrophobic, Green = Polar, Red = Acidic, Blue = Basic, Yellow = Cysteine). The position of canonical OB-fold secondary structures within the sequence are annotated below. Β-strands are labeled (β1–β4) with the intervening loops described by the strands they connect.

In the Zam protein these four residues are also conserved in the L12 loop, though the first lysine has been replaced by an arginine (Figure 3). It is also important to note that it is within the L12 loop of Zam that the conserved Zam cysteines are found. Given the importance of the L12 loop in nucleic acid binding by CSD1 in RNase II-type proteins, it seems likely that these cysteines are involved in the nucleic acid binding process. A possible mechanism of action would be the direct regulation of RNA binding through stabilization of the L12 loop via regulated disulfide formation. Alternatively, other post-translational modifications of cysteines besides disulfides can occur and could serve to enhance or abolish RNA binding at this site.

While the L12 loop is essential for RNA binding in the CSD1 of RNase II, this region is not responsible for RNA binding in all RNase II/R family members. Rrp44, a yeast RNase II/R family member, has been crystalized with RNA [40]. In the Rrp44 structure, RNA enters the protein active site via a side channel formed by the RNB domain and the two CSD domains instead of passing through the central RNA channel utilized by RNA in RNase II [40]. Thus, in Rrp44, a different loop of CSD1 is exposed to RNA to facilitate binding. While the CSD1 of Rrp44 is partially unresolved in the crystal structure, it is apparent that the loop between β-strand 2 and β-strand 3 (L23) contains at least some of the key residues required for binding. In Zam, L23 does not contain any well conserved positively charged or aromatic residues and thus likely does not use L23 as an RNA binding interface.

The mechanism of RNA binding in RNase II/R family members is fairly well understood, but not known for every member. In RNase R it has been suggested that RNA might use either the RNase II-like or the Rrp44-like binding mode [21]. Additionally, since relatively short RNA molecules have been co-crystalized with these proteins, how other regions within the CSD1 domain might interact with RNA remain unknown. Other potential RNA binding residues are conserved within the Zam CSD1, including a tryptophan in loop region L34 and an arginine repeat in L45 (Figure 3). These conserved residues appear to be surface exposed the structural model of Zam (Figure 1D) and could potentially make additional RNA contacts.

### 3.5. CSD1 Cysteine to Serine Mutants Phenocopy Zam Deletion

Proteomics, bioinformatic analysis, computational modeling, and in vitro experimentation suggest that the conserved CSD1 cysteines are involved in Zam function. Therefore, we created and characterized strains with C73 and C79 mutations in vivo. We first created a PCC 7002 Zam deletion line (ΔZ) and a complemented deletion line (ΔZ+) in which PCC 7002 Zam is expressed in a different location in the genome but under its native promoter, as previously described [7]. Additionally, we created three complemented lines (ΔZ+ C73S, ΔZ+ C79S, ΔZ+ DCS) containing mutations that convert one or both native cysteine residues to serine residues.

**Figure 4:**
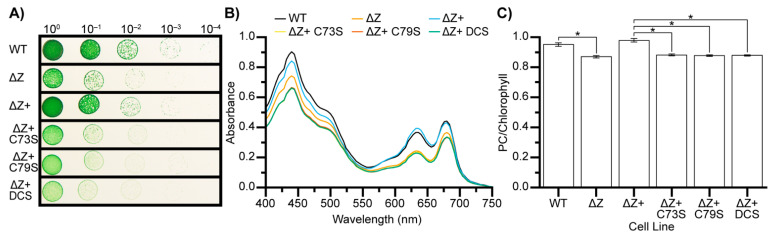
**C73 and C79 are required for Zam function in vivo.** (**A**) Spot plate showing relative growth of cell lines used in this study. As previously reported ΔZ exhibits a mild growth defect. The mutant complemented lines show the same defect. (**B**) Representative absorption spectrums of cell lines used in this study. ΔZ cells and the mutant complement lines show decreased phycocyanin relative to chlorophyll. (**C**) Average ratio of phycocyanin absorption to chlorophyll absorption for each cell line (*n* = 3). Statistical difference determined by Student’s *t*-test at *p* = 0.001 indicated by *.

Spot plate analysis shows that the ΔZ and cysteine mutant lines all exhibit a mild growth defect compared to wild-type (WT) and ΔZ+ (Figure 4A). Additionally, colonies of the ΔZ and cysteine mutants are chlorotic. This chlorotic phenotype is also observed in liquid culture and is caused by a statistically significant decrease in the ratio of phycocyanin relative to chlorophyll in the ΔZ and cysteine mutant lines (Figure 4B,C).

### 3.6. ΔZ and ΔZ+ DCS Exhibit Decreased Synthesis of Phycocyanin

**Figure 5:**
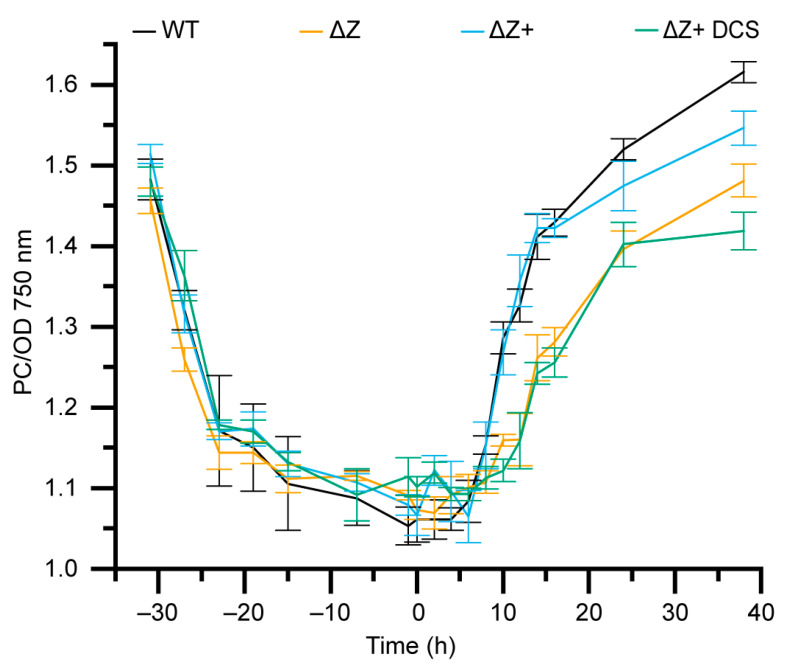
**Time-course of nitrogen depletion and repletion.** Ratio of phycocyanin to optical density 750 nm for cell lines growing under nitrogen starvation for 30 h followed by nitrogen repletion. Nitrate was added to samples at t = 0.

Since the decreased steady state level of phycocyanin in ΔZ and the mutant complement lines could arise from increased phycocyanin degradation, decreased phycocyanin synthesis, or a combination of the two, we sought to isolate and observe both processes. WT, ΔZ, ΔZ+ and ΔZ+ DCS cells were grown in shaking flasks, starved of nitrate to induce the degradation of phycocyanin, and then resupplied with nitrate to reinitiate the synthesis of phycocyanin (Figure 5) [41]. Phycocyanin and chlorophyll levels were monitored spectroscopically. During nitrogen starvation, all four cell lines behave similarly, with a phycocyanin degradation pattern consistent with previous nitrogen starvation experiments [41,42]. However, upon the addition of nitrogen, WT and ΔZ+ exhibit faster initiation, increased rate, and increased yield of phycocyanin synthesis compared to the ∆Z and the cysteine double-mutant (Figure 5). This leads us to conclude that the lower steady state phycocyanin abundance in ΔZ and the cysteine mutants are a result of lower phycocyanin synthesis, and not due to increased phycocyanin turnover.

### 3.7. ΔZ and Cysteine Mutants Exhibit Altered Photosynthetic Energy Transfer

**Figure 6:**
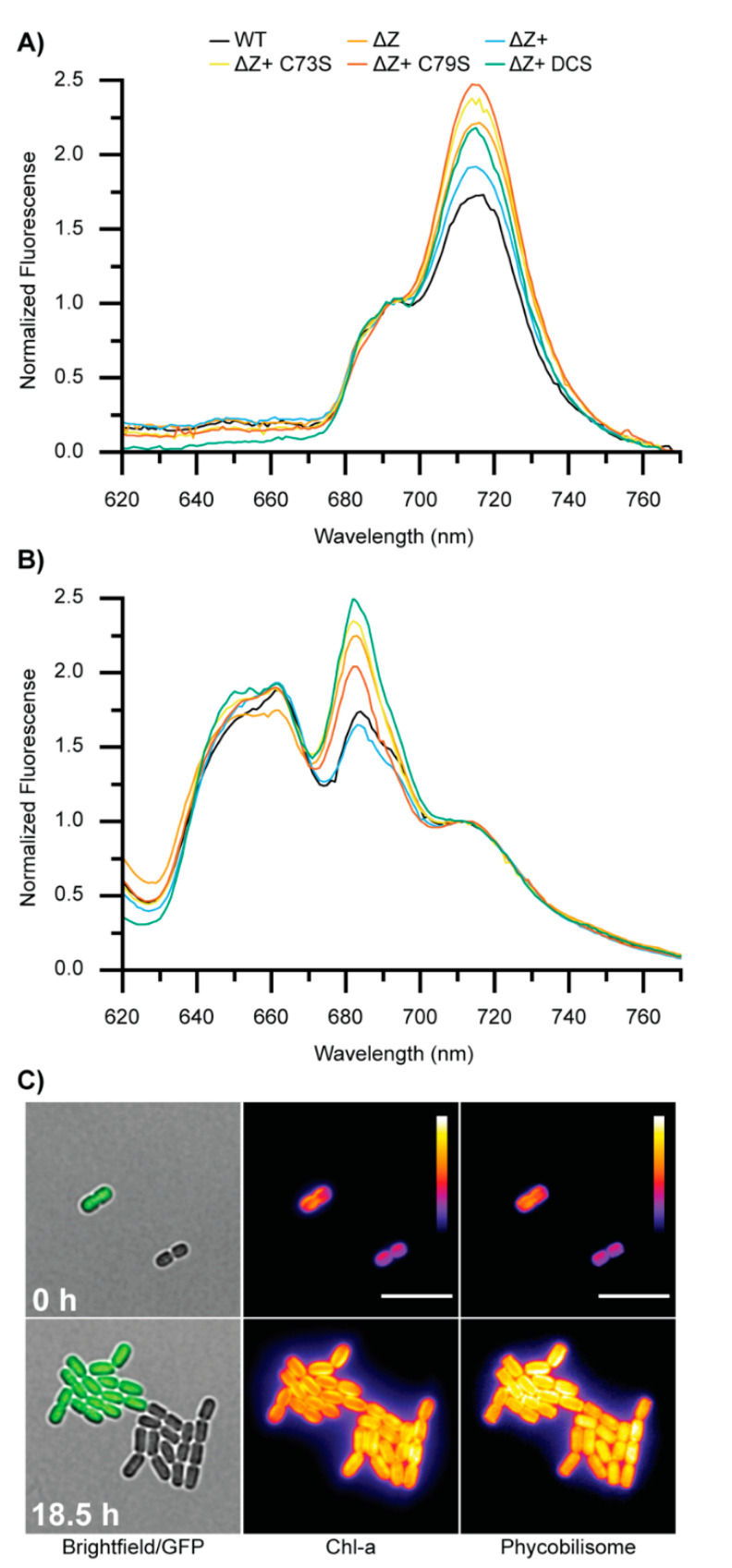
**Bulk 77 K emission spectra and single-cell analysis of growth and fluorescence at room temperature.** (**A**) 77 K fluorescence emission spectra for each cell line produced by chlorophyll excitation at 440 nm normalized to PSII max emission (692 nm). Increased PSI:PSII ratio is observed for ΔZ and mutant complement lines relative to ΔZ and ΔZ+. (**B**) 77 K fluorescence emission spectra produced by phycobilisome excitation at 580 nm normalized to PSI max emission (712 nm). Increased energy transfer to PSII is observed in ΔZ and cysteine mutants. (**C**) Snapshots from time-lapse imaging (Supplementary Movies S1–S3) showing growth and fluorescent properties of ∆Z and control WT-GFP cells under identical conditions for 18.5 h. Specific excitation/emission filters were used to monitor fluorescence of GFP (green), Chl-a (Intensity scale = 177–3000 A.U.; purple-white), and phycobilisomes (Intensity scale = 177–1096 A.U.; purple-white). Scale bar = 10 µm.

The ΔZ and cysteine mutant cell lines showed altered pigment abundance and slower growth rates compared to the controls. Therefore, we reasoned that they could exhibit altered light harvesting or energy transfer phenotypes. We conducted 77 K fluorescence spectroscopy with excitation at either 440 nm to preferentially excite chlorophyll or 580 nm to preferentially excite phycocyanin. Fluorescence emission spectra (600–800 nm) containing peaks originating from terminal emitters of phycocyanin (PC), photosystem II (PSII), and photosystem I (PSI) were then collected (Figure 6). Chlorophyll excitation shows an increased ratio of PSI:PSII in the ∆Z and cysteine mutants compared to WT and ∆Z+ strains (Figure 6A). Phycocyanin excitation shows increased energy transfer from the phycobilisome to PSII in the ΔZ and cysteine mutants compared to WT and ∆Z+. (Figure 6B). Taken together, these results suggest that altered coupling of phycobilisomes and PSII could balance energy flow to compensate for the altered ratio of PSI:PSII. Alternatively, changes to the antenna coupling could affect photosystem stoichiometry.

### 3.8. Comparison of WT and ∆Z Growth and Fluorescence at Single-Cell Resolution

Bulk fluorescence and growth experiments revealed altered pigment profiles and reduced growth capacity of ∆Z compared to the WT. The growth and fluorescence properties of ∆Z and a WT control strain expressing GFP (WT-GFP) were also compared at single(sub)-cellular resolution using long-term time-lapse fluorescence microscopy (Figure 6C and Supplementary Movies S1–S3) on a custom microscope we recently developed for growth and imaging of photosynthetic organisms [28,43]. By confining cells in a 2D layer between the coverslip and pad of growth media solidified with agar, spectrally defined light provided by a solid-state transillumination source (640 nm) can be uniformly applied to the cells (as sole energy source) while simultaneously monitoring the fluorescence dynamics of chlorophyll (Chl-a) and phycobilisomes. Spike-in WT-GFP served as an internal growth control as it has very reproducible growth in addition to producing a distinctive and expected colony morphology at the 16-cell stage [28]. Notably, the ∆Z strain exhibited lower fluorescence in both the Chl-a and phycobilisome channel at the initial timepoint compared to WT-GFP cells at the same cell-cycle stage (Figure 6C upper panel and Figure S2). After 18.5 h growth, the WT-GFP and ∆Z strain both produced 16 cells and typical colony morphologies, further confirming the subtle growth defects observed on solid medium and as shown in Figure 4A. After 18.5 h of growth, the fluorescence intensity of Chl-a and phycobilisomes in ∆Z cells recovered to levels similar to that of WT-GFP. However, there are subtle differences in the appearance and localization of fluorescence between WT-GFP and ∆Z. In particular, WT-GFP exhibits more fluorescence (both Chl-a and phycobilisome) at the periphery of the cell in association with the thylakoid membrane whereas it is more evenly distributed in ∆Z, which could indicate a change in the sub-cellular distribution of phycobilisomes or PSII.

## 4. Discussion

Based on homology and domain architecture, one would presume that the Zam proteins were functional exonucleases, and in many genomes they have been annotated as such. However, closer examination of the protein sequences via multiple sequence alignment shows that the canonical RNB active site is not present in any Zam protein. Without this key region, Zam likely cannot function as a nuclease. The absence of nuclease activity by Zam is consistent with in vivo knockout data from PCC 7002 [7]. In *E. coli*, Rnr, Rnb, and polynucleotide phosphorylase (PNPase) work cooperatively and redundantly to provide the cell with 3′-5′ exonuclease activity. Deletion of any one of those proteins produces only mild phenotypes. However, deletion of PNPase in concert with either Rnr or Rnb is lethal [44,45,46]. In cyanobacteria, deletion of either the PNPase or Rnb in PCC 7002 has been shown to be lethal, while deletion of Zam produces the complex phenotypes outlined in this and other works [7]. Our data are consistent with the idea that Zam is not functional as a nuclease and cannot provide redundancy to the RNA degradation system. Indeed, purified PCC 7002 Zam (Figure S1) did not exhibit any nuclease or RNA binding activity when incubated with several purified Rnb substrates (data not shown). As our purified protein is prone to aggregation, any interpretation of these negative results must be done with an abundance of caution. However, they are consistent with the bioinformatic and in vivo findings.

If Zam proteins are not functioning as nucleases, they must be exerting their influence on the cell through another mechanism. The conservation of the cold shock and S1 domains in Zam proteins indicates they may represent the major functional domains of the protein. Our work indicates that the conserved cysteines in the CSD1 of Zam undergo redox modulation and are essential for in vivo function, with mutagenesis of the redox active cysteines in CSD1 being sufficient to produce phenotypes identical to that of gene knockout. We hypothesize that the conserved cysteines in CSD1 are subject to control by the cellular redox state and that locking them in the permanently reduced state renders Zam nonfunctional. CSD1 is canonically an RNA binding domain in RNase II/R proteins, and it is possible that cellular redox state is modulating the ability of Zam to bind RNA targets via the disulfide modulation of CSD1 [39]. However, a recent study shows that an RNase II/R protein (identified here as Zam) from *Anabaena* sp. PCC 7120 interacts with and enhances the activity of RNase E, the major degradosome enzyme, via its cold-shock and S1 domains [47]. This finding prompts us to propose a different model in which Zam binding to RNase E under oxidizing, but not reducing, conditions increases RNA turnover rates. Based on current data, it is unknown whether the interaction between Zam and RNase E is mediated fully or in part by RNA interactions, but as our analysis shows CSD1 maintains the canonical RNA binding residues, an RNA mediated binding modality is a distinct possibility.

A role for Zam in regulating RNA turnover via interaction with the degradosome would be consistent with both our analysis and the literature. The original phenotype of *zam* deletion in PCC 6803 was a tolerance of carbonic anhydrase inhibitors and we see this phenotype to a lesser degree in PCC 7002 (Figure S3) [3,4]. The authors of the papers characterizing the PCC 6803 mutant hypothesized that *zam* encoded a transporter that affected the access of the inhibitor to the carbonic anhydrase [4]. While this explanation is unlikely based on the evolutionary roots of Zam, it is simple to imagine a change in global RNA degradation rates affecting membrane protein abundance and changing transport. A similar phenomenon could explain the alteration of photosystem or phycobilisome complex abundance that we see in our *zam* deletion or mutant strains using bulk and single-cell analysis.

This work provides new insight into the RNase II/R family of cyanobacteria. We classify Zam proteins as non-functional nucleases and show that they have a role in controlling diverse phenotypes including growth rate, pigment biosynthesis, and the PSI:PSII ratio. The ability of Zam to function in vivo is dependent upon redox active cysteines located in CSD1, and this highly conserved region is almost certainly responsible for controlling the function of Zam. While further experimentation is needed, a model where the redox state of Zam modulates both the interaction with and subsequent activation of RNase E seems likely based on our work and the literature.

## Data Availability

The authors confirm that the data supporting the findings of this study are available within the article and Supplementary Materials. Additional materials and strains available upon reasonable request to Jeffrey C. Cameron (jeffrey.c.cameron@colorado.edu).

## Supplementary Materials

The following supporting information can be downloaded at: https://www.mdpi.com/article/10.3390/microorganisms10051055/s1, This manuscript contains the following supplemental information; Supplemental Table S1. Taxid and reference IDs for organisms and sequences used in this study; Supplemental Table S2. Strains used in this study; Supplemental Table S3. Plasmids used in this study; Supplemental Table S4. DNA oligonucleotides used in this study; Supplemental Figure S1. Purification of PCC 7002 Zam from *E. coli*; Supplemental Figure S2. Image of ∆Z and WT-GFP using fluorescence microscopy; Supplemental Figure S3. Growth of WT and ∆Z in presence of acetazolamide; Supplemental Movie S1. Time-lapse video of ∆Z and WT-GFP with brightfield and GFP channels; Supplemental Movie S2. Time-lapse video of ∆Z and WT-GFP with chlorophyll channel; Supplemental Movie S3. Time-lapse video of ∆Z and WT-GFP with phycobilisome channel.
