# Supplementary material for: Zam Is a Redox-Regulated Member of the RNB-Family Required for Optimal Photosynthesis in Cyanobacteria
Source: Microorganisms. 2022 May 20;10(5):1055. doi: 10.3390/microorganisms10051055 (PMC9145284; doi:10.3390/microorganisms10051055)
Supplement: Supplementary file 1 [file microorganisms-10-01055-s001.zip › Figure S1.pdf]

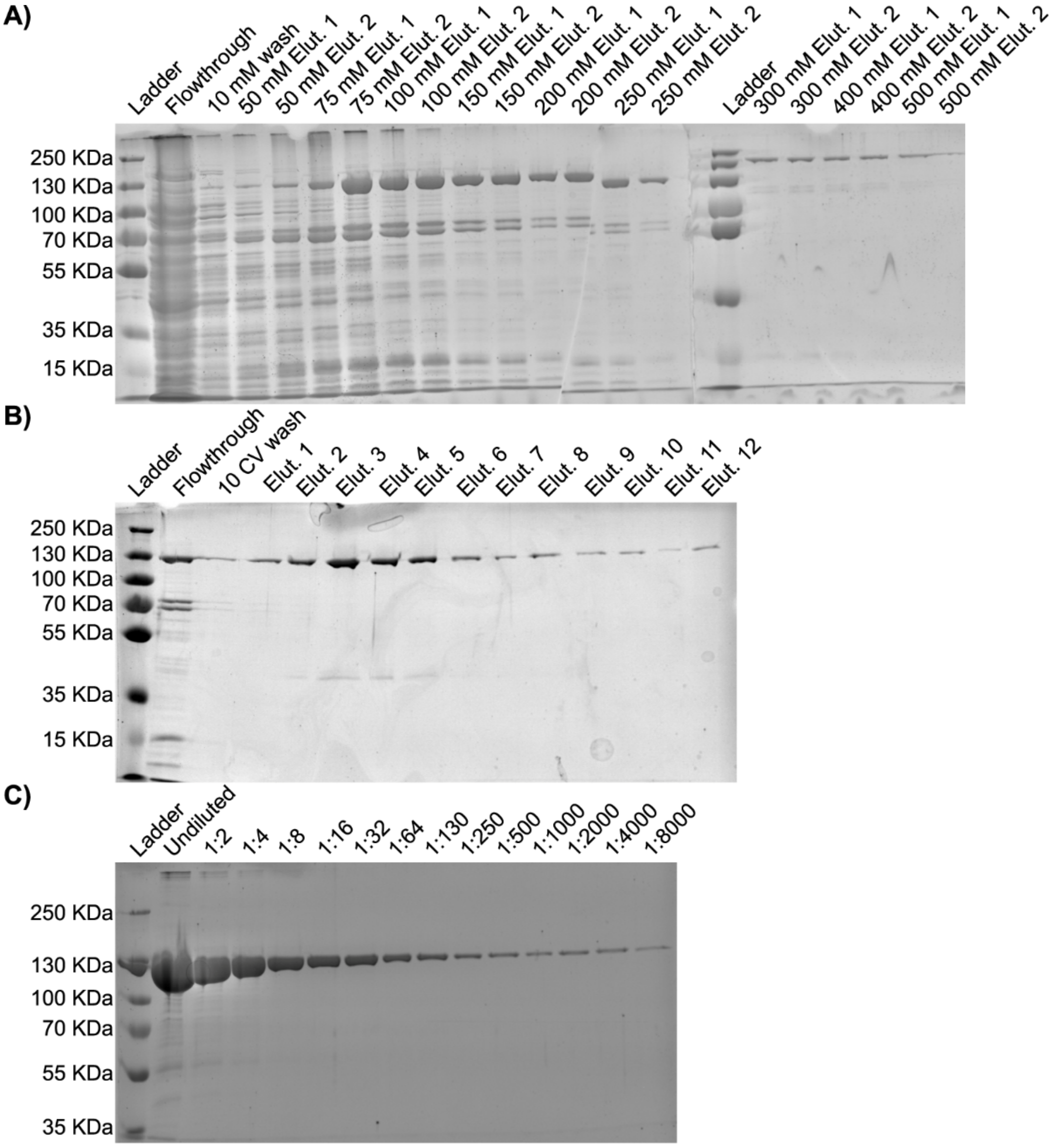

Figure S1. Purification of PCC 7002 Zam from *E. coli*. WT Zam purification example. A) SDS-PAGE showing lysis and Ni-NTA purification. Concentration of imidazole used in wash/elution is shown. All samples from 75 mM imidazole #2 on were pooled and used for amylose resin purification. B) SDS-PAGE gel showing amylose resin purification. Samples from elution #2 on were pooled and concentrated using a 100 kDa spin concentrator. C) SDS-Page gel showing dilution series of final purified protein. Concentration in lane #1 is approximately 500 μM.
