# Supplementary material for: Zam Is a Redox-Regulated Member of the RNB-Family Required for Optimal Photosynthesis in Cyanobacteria
Source: Microorganisms. 2022 May 20;10(5):1055. doi: 10.3390/microorganisms10051055 (PMC9145284; doi:10.3390/microorganisms10051055)
Supplement: Supplementary file 1 [file microorganisms-10-01055-s001.zip › Figure S2.pdf]

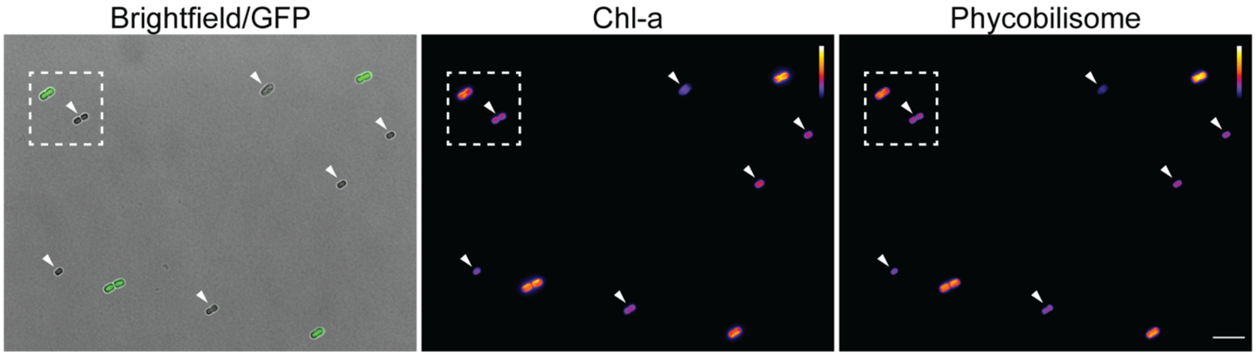

Figure S2. Image of ΔZ and WT-GFP cells using fluorescence microscopy. ΔZ cells were mixed with WT cells expressing GFP (to distinguish between strains) and applied to a pad of solidified growth medium and imaged using transmitted light (brightfield) or specific excitation/emission filters to quantify fluorescence of GFP (green), chlorophyll-a (Intensity scale = 177-3000 A.U.; purple-white), and phycobilisomes (Intensity scale = 177-1096 A.U.; purple-white). White arrows indicate ΔZ cells. Cells shown in Fig. 6 and Supplemental Movie S1-S3 are boxed. Scale bar = 10 μm.
