# Supplementary material for: Zam Is a Redox-Regulated Member of the RNB-Family Required for Optimal Photosynthesis in Cyanobacteria
Source: Microorganisms. 2022 May 20;10(5):1055. doi: 10.3390/microorganisms10051055 (PMC9145284; doi:10.3390/microorganisms10051055)
Supplement: Supplementary file 1 [file microorganisms-10-01055-s001.zip › Figure S3.pdf]

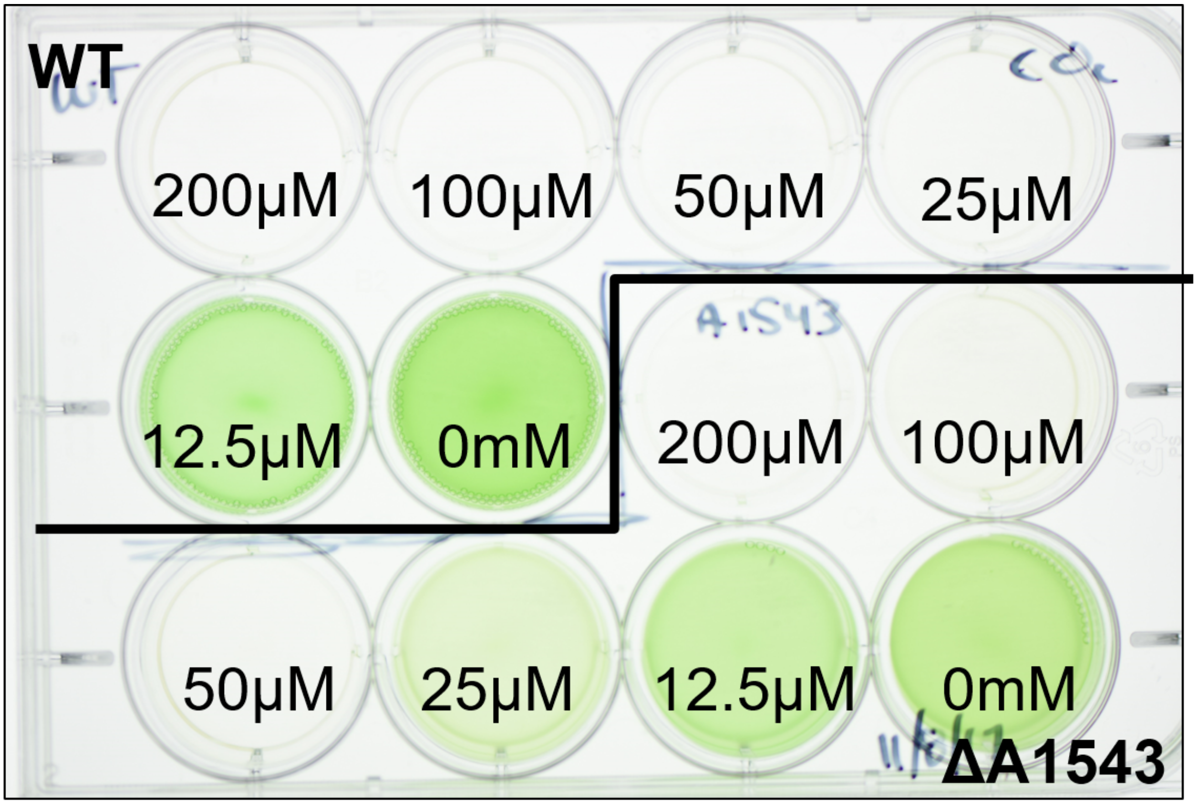

Figure S3. Growth of WT and ΔZ in presence of acetazolamide. Representative experiment of wildtype and ΔZ cells grown with various concentrations of acetazolamide. ΔZ cells show ~2-fold increased tolerance to acetazolamide under all conditions tested.
