# Supplementary material for: Zam Is a Redox-Regulated Member of the RNB-Family Required for Optimal Photosynthesis in Cyanobacteria
Source: Microorganisms. 2022 May 20;10(5):1055. doi: 10.3390/microorganisms10051055 (PMC9145284; doi:10.3390/microorganisms10051055)
Supplement: Supplementary file 1 [file microorganisms-10-01055-s001.zip › Table S1.pdf]

Table S1. Taxid and reference IDs for organisms and sequences used in this study.

Organism	Type	Taxid ID	PCC 7002 Zam (WP_012307157.1) Homolog ID	PCC 7002 Rnb (WP_012306205.1) Homolog ID
Acaryochloris mariana MBIC11017	Cyanobacteria	329726	WP_012164530	WP_012165064
Anabaena sp. PCC 7120	Cyanobacteria	103690	WP_096637251	WP_010998583
Arthrospira platensis NIES-69	Cyanobacteria	696747	WP_006616719	WP_035737129
Cyanobacterium sp. UCYN-A	Cyanobacteria	713887	WP_041487771	WP_012954168
Cyanothece sp. BH68	Cyanobacteria	43989	WP_009543092	WP_009545481
Gleobacter violaceus PCC 7421	Cyanobacteria	251221	WP_011141448	WP_011141615
Micryocystis aeruginosa NIES-843	Cyanobacteria	449447	WP_012265538	WP_012267238
Prochlorococcus Marinus pastoris CCMP 1986	Cyanobacteria	59919	WP_011132481	WP_011132502
Synechococcus elongatus PCC 7942	Cyanobacteria	1140	WP_011378237	WP_011242737
Synechocystis sp. PCC 6803	Cyanobacteria	1148	WP_010871910	WP_010871832
Thermosynechococcus elongatus BP-1	Cyanobacteria	197221	WP_011057710	WP_011056499
Trichodesmium erythraeum IMS101	Cyanobacteria	203124	WP_011611172	WP_011613117
Epithemia turgida endosymbiont	Diatom	718217	WP_044107244	WP_044107596
Paulinella chromatophora	Amoeba	39717	YP_002049468	YP_002048710
Chlamydomonas reinhardtii (nuclear)	Algae	3055	None	XP_001693304
Chlamydomonas reinhardtii (chloroplastic)	Algae	3055	None	ABO20871.2
