# Supplementary material for: Zam Is a Redox-Regulated Member of the RNB-Family Required for Optimal Photosynthesis in Cyanobacteria
Source: Microorganisms. 2022 May 20;10(5):1055. doi: 10.3390/microorganisms10051055 (PMC9145284; doi:10.3390/microorganisms10051055)
Supplement: Supplementary file 1 [file microorganisms-10-01055-s001.zip › Table S2.pdf]

Table S2. Strains used in this study.

Name	Number	Description	Resistance	Reference
WT	-	Wild-type *Synechococcus sp*. PCC 7002	-	-
WT GFP		WT cells transformed by EBJc0025	Gentamicin	This Work
ΔZ	scJC0122	WT cells transformed by pJCC0254	Spectinomycin	Cameron 2015
ΔZ+	scJC0254	ΔZ cells transformed with pJCC257	Kanamycin	Cameron 2015
ΔZ+ C73S	-	ΔZ cells transformed with pPET046	Kanamycin	This Work
ΔZ+ C79S	-	ΔZ cells transformed with pPET047	Kanamycin	This Work
ΔZ+ DCS	-	ΔZ cells transformed with pPET048	Kanamycin	This Work
MBP-Zam-His Expression	sJC0482	*E. coli* BL21 transformed with pPET095	Kanamycin	This Work
