# Supplementary material for: Zam Is a Redox-Regulated Member of the RNB-Family Required for Optimal Photosynthesis in Cyanobacteria
Source: Microorganisms. 2022 May 20;10(5):1055. doi: 10.3390/microorganisms10051055 (PMC9145284; doi:10.3390/microorganisms10051055)
Supplement: Supplementary file 1 [file microorganisms-10-01055-s001.zip › Table S3.pdf]

Table S3. Plasmids used in this study.

Number	Description	Genomic Location	Reference
pJCC0254	pUC19-ΔglpK:KmR-A1543	*zam*	Cameron et al. 2015 [7]
pJCC0257	pUC19-ΔglpK:KmR-*zam*	*glpK*	Cameron et al. 2015 [7]
pPET046	pUC19-ΔglpK:KmR-*zam*; C73G	*glpK*	This Work
pPET047	pUC19-ΔglpK:KmR-*zam*; C79G	*glpK*	This Work
pPET048	pUC19-ΔglpK:KmR-*zam*; C73G & C79R	*glpK*	This Work
EBJc0025	pUC19-ΔglpK:GmR_P_{ccmK2}-sfGFP	*glpK*	This Work
pPET095	pET28B MBP-Zam-6xHIS	-	This Work
