# Supplementary material for: Zam Is a Redox-Regulated Member of the RNB-Family Required for Optimal Photosynthesis in Cyanobacteria
Source: Microorganisms. 2022 May 20;10(5):1055. doi: 10.3390/microorganisms10051055 (PMC9145284; doi:10.3390/microorganisms10051055)
Supplement: Supplementary file 1 [file microorganisms-10-01055-s001.zip › Table S4.pdf]

Table S4. DNA oligonucleotides used in this study.

Number	Description	Sequence 5' -> 3'	Reference
PET_103F	A1543 internal primer for sequencing	ggggtgaggtgaatttcatcctc	This Work
PET_104R	A1543 internal primer for sequencing	gatttactgcgccaatttcccg	This Work
PET_105F	A1543 internal primer for sequencing	gagttttaaaatgcgctgcatcacc	This Work
PET_106R	A1543 internal primer for sequencing	gacggacatgacaaagatcctgaactatc	This Work
PET_107F	A1543 Upstream Flanking Region	catcagctgagtcatagacgggtg	This Work
PET_108R	A1543 Downstream Flanking Region	gtttcactttactttctaacgaaaaggatagttttgc	This Work
PET_109F	A1543 C73F Mutation Primer Forward	caaaacagaaacccttactcgaaccccgtagctttgcctcgacc	This Work
PET_110R	A1543 C73F Mutation Primer Reverse	tacggggttcgagtaagggtttctgttttgcg	This Work
PET_111F	A1543 C79G Mutation Primer Forward	caaaaccgaaacccttactcgaacaccgtagc	This Work
PET_112R	A1543 C79G Mutation Primer Reverse	tacggtgttcgagtaagggtttcggttttgcgatccaggattc	This Work
PET_114R	A1543 C73G & C79G Mutation Primer Reverse	tacggggttcgagtaagggtttcggttttgcgatccaggattc	This Work
PET_115F	A1543 C73G & C79G Mutation Primer Reverse	caaaaccgaaacccttactcgaaccccgtagctttgcctcgacc	This Work
